# Biomarkers of subclinical atherosclerosis in patients with psoriasis

**DOI:** 10.1038/s41598-021-00999-9

**Published:** 2021-11-02

**Authors:** Hannah Kaiser, Xing Wang, Amanda Kvist-Hansen, Martin Krakauer, Peter Michael Gørtz, Benjamin D. McCauley, Lone Skov, Christine Becker, Peter Riis Hansen

**Affiliations:** 1 Department of Cardiology, Copenhagen University Hospital - Herlev and Gentofte, Copenhagen, Denmark; 2 Department of Dermatology and Allergy, Copenhagen University Hospital - Herlev and Gentofte, Copenhagen, Denmark; 3 Department of Clinical Medicine, University of Copenhagen, Copenhagen, Denmark; 4 Division of Clinical Immunology, Department of Medicine, Icahn School of Medicine at Mount Sinai, New York, NY USA; 5 Department of Clinical Physiology and Nuclear Medicine, Herlev and Gentofte Hospital, Hellerup, Denmark; 6 Department of Clinical Physiology and Nuclear Medicine, Bispebjerg and Frederiksberg Hospital, University Hospital, Copenhagen, Denmark; 7 Department of Genetics and Genomic Sciences, Icahn School of Medicine at Mount Sinai, New York, NY USA

**Keywords:** Skin diseases, Cardiology, Biomarkers

## Abstract

Psoriasis is linked with increased risk of cardiovascular disease (CVD) that is underestimated by traditional risk stratification. We conducted a large-scale plasma proteomic analysis by use of a proximity extension assay in 85 patients with a history of moderate-to-severe psoriasis with or without established atherosclerotic CVD. Differentially expressed proteins associated with CVD were correlated with subclinical atherosclerotic markers including vascular inflammation determined by ^18^F-fluorodeoxyglucose positron emission tomography/computed tomography, carotid intima-media thickness (CIMT), carotid artery plaques, and coronary artery calcium score (CCS) in the patients without CVD and statin treatment. We also examined the association between the neutrophil-to-lymphocyte ratio (NLR) and subclinical atherosclerosis. In unadjusted analyses, growth differentiation factor-15 (GDF-15) levels and NLR were increased, while tumor necrosis factor (TNF)-related activation-inducing ligand (TRANCE) and TNF-related apoptosis-induced ligand (TRAIL) levels were decreased in patients with established CVD compared to those without CVD. Among patients with psoriasis without CVD and statin treatment, GDF-15 levels were negatively associated with vascular inflammation in the ascending aorta and entire aorta, and positively associated with CIMT and CCS. NLR was positively associated with vascular inflammation in the carotid arteries. Our data suggest that circulating GDF-15 levels and NLR might serve as biomarkers of subclinical atherosclerosis in patients with psoriasis.

## Introduction

Psoriasis is a chronic systemic inflammatory disease affecting approximately 3% of the adult population^1,2^. The condition is associated with increased risk of cardiovascular disease (CVD), e.g. myocardial infarction (MI) and stroke^3^, as well as a reduced lifespan often caused by CVD^4^. The risk of CVD in these patients is underestimated by traditional CVD risk stratification and national guidelines for CVD risk stratification therefore highlight the importance of integrating psoriasis in the clinical assessment of CVD risk^5–8^.

^18^F-fluorodeoxyglucose (FDG) uptake primarily by macrophages in the arterial wall detected by positron emission tomography/computed tomography (PET/CT) is a valuable method for assessment of vascular inflammation^9^, and increased FDG uptake in the aorta and carotid arteries is associated with coronary plaque burden^10^ and predictive of future CVD^11^. Increased risk of atherosclerotic CVD in patients with psoriasis may be driven, in part, by increased systemic inflammation^1^ and PET/CT and other imaging modalities have demonstrated increased subclinical atherosclerotic disease, e.g. increased vascular inflammation, and augmented coronary artery calcification and carotid intima-media thickness (CIMT), respectively, in these patients^12–14^.

There is a continuing need for new biomarkers to assess the risk of CVD in psoriasis and, by inference, the magnitude of subclinical CVD in these patients. In this regard, many studies have used a selective (‘candidate biomarker’) approach and only examined a few biomarkers^15–18^. By using high throughput technologies, it is possible to evaluate an extensive number of proteins simultaneously, which facilitates novel biomarker discovery. Indeed, a recent study with a multiplex proximity extension assay found that circulating levels of chemokine ligand 20 (CCL20) predicted vascular endothelial inflammation determined by endothelial cell inflammatory transcripts in patients with psoriasis^19^. Also, increased neutrophil-to-lymphocyte ratio (NLR), which reflects a heightened state of systemic inflammation, is increased in patients with psoriasis and has been shown to be a predictor of CVD and subclinical atherosclerosis^20–22^. In the present study, we used the proximity extension assay to investigate potential plasma protein biomarkers of subclinical atherosclerotic CVD assessed by ^18^F-FDG-PET/CT, coronary artery calcium score (CCS), and carotid artery ultrasound variables in patients with a history of moderate-to-severe psoriasis. We also examined the link between NLR and subclinical CVD in these patients.

## Methods

### Study population

Patients with moderate-to-severe psoriasis defined as treated with systemic anti-psoriatic treatment or a Psoriasis Area and Severity Index (PASI) above 10, aged ≥ 30 years were primarily recruited from a single tertiary dermatology university hospital clinic in Copenhagen, Denmark. Study design and inclusion- and exclusion criteria have been reported previously^23^. Approximately half of the patients had prior atherosclerotic CVD, including stroke, MI, peripheral artery disease and/or coronary artery revascularization > 6 months before inclusion. In addition, approximately half of the overall cohort received systemic anti-psoriatic therapy. All patients were interviewed and the severity of psoriasis at inclusion was assessed with the PASI. The study was conducted in compliance with the Declaration of Helsinki and approved by the ethical committee of the Capital Region in Denmark and the local data protection agency. All participants provided informed consent before inclusion.

### Blood samples and targeted proteomics analyses

Blood from all patients was collected and analyzed for lipid profile, leukocytes, and high-sensitivity C-reactive protein (hs-CRP) at the Department of Clinical Biochemistry, Herlev and Gentofte Hospital, Denmark. NLR was calculated by dividing the absolute neutrophil count with the absolute lymphocyte count.

Plasma was stored at − 80 °C until proteomic analyses were performed using the Olink Proseek^®^ multiplex assay (Olink Bioscience, Uppsala, Sweden) that employs proximity extension assay technology to detect proteins in plasma samples^24^. In brief, the proximity extension assay utilizes pairs of oligonucleotide-labelled antibodies that bind to their specific target protein. Upon binding in proximity, the oligonucleotides hybridize whereby unique DNA barcodes for the respective proteins are created, and these are subsequently amplified and quantified by real-time PCR. We used the pre-designed Olink multiplex panels Inflammation, Cardiovascular II, and Cardiovascular III, with a total of 92 proteins in each panel^25^. The Olink platform provides normalized protein expression (NPX) values reported on a log_2_ scale where high NPX values correspond to high protein concentrations but are not absolute concentration measurements. There were 10 overlapping proteins which were analyzed twice in these panels. Patient samples that did not pass the quality control were removed. Also, proteins were removed if > 40% of the samples were below the lower limit of detection and thereby excluded from further analyses.

### ^18^F-FDG-PET/CT imaging and analysis

Whole body ^18^F-FDG-PET/CT scan was performed with a GE Discovery 710 scanner (General Electric Medical Systems, Milwaukee, WI, USA) using the proprietary Q.Clear™ PET reconstruction algorithm. The scan was performed within 14 days after the blood samples were collected. After overnight fasting, 3.5 MBq (0.09 mCi)/kg FDG was administered intravenously and imaging was performed after 120 min. An unenhanced CT scan was used for anatomic co-registration and attenuation correction. Image analyses were performed in accord with established methodology^26^ by use of the MIM 6.9.2 software (MIM Software Inc., Cleveland, OH, USA). Measurements for the aorta and carotid arteries were made on axial slices every 3 mm along the full anatomical course of the respective arteries by manually drawing regions-of-interests (ROIs). For the carotid arteries, only slices with a clearly visible anatomical correlate were included. The maximum standardized uptake value (SUV) of FDG was quantified in each slice and blood-corrected values were used by dividing SUV_max_ with SUV_mean_ in a 5 mL ROI in the vena cava superior, resulting in maximum target-to-background ratio (TBR_max_) values. The average maximum target-to-background ratio (TBR_max_) for the aorta was reported for the ascending aorta and the entire aorta, respectively, and mean TBR_max_ was calculated from all slices of both carotid arteries.

### Coronary artery calcium score and carotid artery ultrasound imaging

CCS was determined by ECG-gated low dose CT scan. Siemens SyngoVia software VB40 (Siemens Healthcare, Erlangen, Germany) was used to calculate CCS according to established methodology using a combined Agatston score^27^. CCS was not determined in patients with prior coronary artery revascularization.

Ultrasound imaging of the carotid arteries was performed by using an Affiniti 70G ultrasound machine with a 5–12 MHz linear array transducer (Philips Ultrasound Inc., Bothell, WA, USA) and use of Philips Q-App IMT software (version 3.03). During diastole, CIMT and presence of carotid artery plaques were measured according to accepted methodology^28^. The mean of CIMT from the right and left carotid arteries was calculated.

### Statistical analyses

Baseline characteristics are presented as means ± SDs for parametric variables and medians (IQRs) for non-parametric variables. Histograms and Q–Q plots were used to examine normality distributions. To investigate differences between groups, Student’s t-test and Wilcoxon–Mann–Whitney test were used for parametric and non-parametric data, respectively, and Chi-square test was used for categorical variables.

Statistical analysis of proteomic data was performed without any further normalization steps. Data distribution of NPX values was assessed by use of density- and box-plots, and multidimensional scaling plots were used to visualize patterns and detect potential outliers. Analyses of NPX values in patients with or without CVD was performed by the OlinkAnalyze package provided by Olink and the limma-trend framework from the limma package^29^. The models included CVD as a factor with individual protein NPX values as dependent variables, as well as models with adjustments for sex, age, body mass index (BMI), systemic anti-psoriatic treatment, and statin treatment. p-values were adjusted for multiple testing using the false discovery rate (FDR) by the Benjamini–Hochberg method^30^. Protein changes with a FDR < 0.05 (adjusted p-value) were considered statistically significant representing differentially expressed proteins. Associations between differentially expressed proteins, subclinical atherosclerotic markers and clinical parameters were assessed in patients without CVD and statin treatment (n = 36) by a pairwise correlation matrix with Pearson correlation tests.

Due to the skewed distribution of CCSs we analyzed the distribution of the differentially expressed proteins in patient groups with CCS = 0, CCS > 0–100, and CCS > 100, respectively^31^. These analyses of CCS and carotid plaques (no plaques, plaques in one artery, or plaques in both arteries) were performed by analysis of variance (ANOVA) and further post hoc analysis by pairwise comparisons using Tukey test. Following ANOVA, the NPX values were transformed from log_2_ scale to log_1.1_ scale and the risks of having CCS > 100 or carotid plaques in both carotid arteries were modelled by ordinal logistic regression with a linear effect of the logarithmically transformed proteins. Therefore, calculated odds ratios (ORs) indicated effects of a 10% increase in the respective protein levels. Ordinal logistic regression was also used to examine associations between NLR and CCS or carotid plaques, respectively in patients without prior CVD and statin treatment. In addition, multivariable linear regression analyses adjusted for statin therapy, and CV risk factors including sex, age, BMI, systolic blood pressure, smoking, diabetes mellitus and total cholesterol levels were used, as appropriate. The America College of Cardiology/American Heart Association (ACC/AHA) risk score^32^ that measures the 10-year risk of CVD was calculated for patients without CVD and statin treatment, and models including linear- and multivariable regressions, and ordinal logistic regression with adjustments, respectively, were used to further investigate the predictive value of GDF-15 and NLR for the measures of subclinical CVD. p-values < 0.05 were considered statistically significant. All statistical analyses were performed with R version 4.03 (R Foundation for Statistical Computing, Vienna, Austria).

## Results

### Baseline characteristics

A total of 86 patients were included, but one patient was excluded whose plasma sample did not pass quality control for the proteomic analyses. Therefore, the study comprised a total of 85 patients with a history of moderate-to-severe psoriasis (Table 1). Of these, the mean (SD) age was 59.1 (11.0) years and 71.8% were men. Patients with CVD were more frequently diagnosed with diabetes (35.9 vs. 15.2%; p = 0.028), treated with statins (79.5 vs. 21.7%; p < 0.001), and had lower total cholesterol (3.88 [0.78] vs. 4.97 [0.80]; p < 0.001) and low-density lipoprotein cholesterol (1.92 [0.67] vs. 2.38 [0.74]; p < 0.001) levels, respectively. Median PASI of all patients was 3.6, reflecting that approximately half of all patients (51.8%) with a history of moderate-to-severe psoriasis were treated with systemic anti-psoriatic agents at the time of study inclusion. NLR was increased among patients with CVD compared with those without CVD (2.85 [1.40] vs. 2.32 [1.03]; p = 0.046), and the results remained significant after adjustments for sex, age, BMI, smoking, diabetes mellitus, systolic blood pressure and total cholesterol levels (p = 0.017). A total of 82 patients underwent ^18^F-FDG-PET/CT (n = 3 were excluded due to drop outs [n = 2] or blood glucose > 11.1 mmol/L [n = 1]) and FDG uptakes in the carotid arteries (n = 80 due to exclusion of 2 patients without clearly defined anatomical correlate), the entire aorta, and the ascending aorta, respectively, quantified by TBR_max_ and showed no differences between patients with or without CVD. CCS was determined in 61 patients (n = 24 excluded due to prior coronary artery revascularization) and patients with CVD had higher CCSs compared with patients without CVD. Ultrasound imaging of the carotid arteries was performed in 84 patients (n = 1 was excluded due to drop out) and CIMT was similar between patients with or without CVD while carotid artery plaques occurred more frequently in those with CVD (Table 1).

**Table 1:** Study patient characteristics.

	Entire population (n = 85)	CVD (n = 39)	No CVD (n = 46)	p-value
Sex, male, n (%)	61 (71.8)	28 (71.8)	33 (71.7)	0.996
Age (years)	59.1 ± 11.0	60.2 ± 8.9	58.2 ± 12.4	0.402
BMI (kg/m^2^)	30.1 ± 5.6	30.0 ± 5.3	30.2 ± 5.9	0.883
PASI*	3.6 (1.2–11.0)	4.0 (2.2–9.0)	3.0 (0–11.0)	0.351
PsA, n (%)	21 (24.7)	10 (25.6)	11 (23.9)	0.854
Medically treated hypertension, n (%)	38 (44.7)	23 (59.0)	15 (32.6)	0.015
Medically treated diabetes, n (%)	21 (24.7)	14 (35.9)	7 (15.2)	0.028
Systemic anti-psoriatic treatment, n (%)	44 (51.8)	20 (51.53)	24 (52.2)	0.935
Statin treatment, n (%)	41 (48.2)	31 (79.5)	10 (21.7)	< 0.001
Smoking, present or previous, n (%)	61 (71.8)	31 (79.5)	30 (65.2)	0.145
HbA1c (mmol/mol)	36.0 (34.0–41.0)	37.0 (35.0–47.5)	35.0 (33.3–37.0)	0.026
Total cholesterol (mmol/L)	4.47 ± 0.96	3.88 ± 0.78	4.97 ± 0.80	< 0.001
LDL-C (mmol/L)	2.41 ± 0.84	1.92 ± 0.67	2.83 ± 0.74	< 0.001
HDL-C (mmol/L)	1.24 ± 0.38	1.25 ± 0.42	1.22 ± 0.34	0.742
Triglycerides (mmol/L)	1.66 (1.10–2.50)	1.48 (1.10–1.84)	1.98 (1.20–2.77)	0.042
Blood glucose (mmol/L)	6.28 ± 1.29	6.30 ± 1.44	6.26 ± 1.16	0.891
hs-CRP (mg/L)	1.40 (0.67–3.82)	1.01 (0.68–3.56)	1.90 (0.67–3.77)	0.669
Neutrophils (10^9^/L)	4.24 ± 1.40	4.49 ± 1.30	4.03 ± 1.46	0.130
Lymphocytes (10^9^/L)	1.85 ± 0.66	1.79 ± 0.71	1.90 ± 0.61	0.455
NLR	2.56 ± 1.23	2.85 ± 1.40	2.32 ± 1.03	0.046
Carotid arteries (TBR_max_)	1.71 ± 0.36	1.70 ± 0.35	1.72 ± 0.38	0.866
Entire aorta (TBR_max_)	2.26 ± 0.38	2.20 ± 0.36	2.31 ± 0.40	0.164
Ascending aorta (TBR_max_)	2.45 ± 0.45	2.36 ± 0.42	2.52 ± 0.46	0.113
CIMT (mm)	0.72 ± 0.13	0.75 ± 0.11	0.70 ± 0.14	0.063
**Carotid artery plaques, n (%)**				0.013
No plaques	35 (41.7)	11 (28.9)	24 (52.2)	
Plaques in one artery	17 (20.2)	6 (15.8)	11 (23.9)	
Plaques in both arteries	32 (38.1)	21 (55.3)	11 (23.9)	
**CCS, median (IQR)**	20.5 (1.0–283.0)	283.0 (30.0–652.0)	4.5 (0.0–123.5)	0.002
CCS = 0	15 (24.6)	1 (5.9)	14 (31.8)	0.033
CCS > 0–100	24 (39.3)	6 (35.3)	18 (40.9)	
CCS > 100	22 (36.1)	10 (58.8)	12 (27.3)	

Parametric data are reported as means ± SDs and non-parametric data are medians (IQRs).

*BMI* body mass index, *PASI* psoriasis area and severity index, *PsA* psoriatic arthritis, *CVD* cardiovascular disease, *HbA1c* glycated haemoglobin, *LDL-C* low-density lipoprotein cholesterol, *HDL-C* high-density lipoprotein cholesterol, *hs-CRP* high-sensitivity C-reactive protein, *NLR* neutrophil-to-lymphocyte ratio, *TBR*_*max*_ maximum target-to-background ratio, *CIMT* carotid intima-media thickness, *CCS* coronary artery calcium score.

*Approximately half of patients in each group were treated with systemic anti-psoriatic agents (see text).

### Differentially expressed proteins

**Figure 1:**
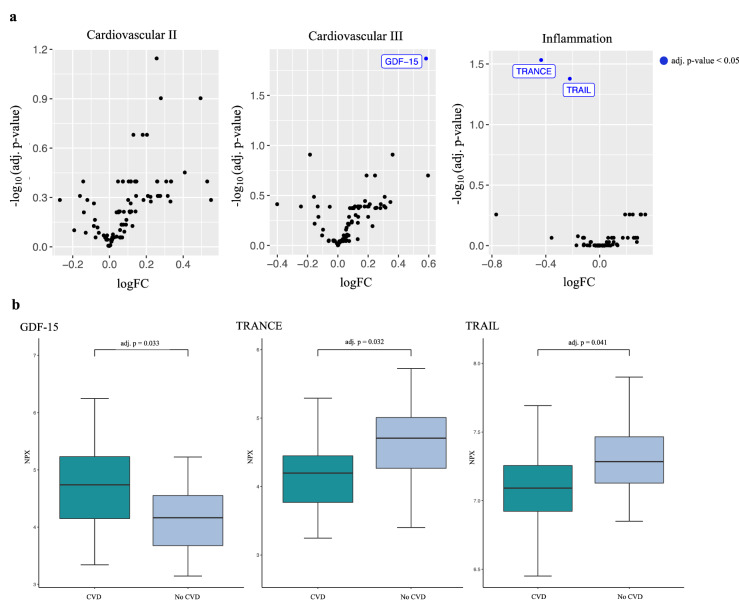
Differentially expressed proteins associated with cardiovascular disease in patients with psoriasis. (**a**) Volcano plots and (**b**) box plots for differentially expressed proteins assessed by proteomic panels Cardiovascular II and III, and Inflammation (Olink Bioscience, Uppsala, Sweden) in patients with psoriasis with or without cardiovascular disease (n = 85). *GDF-15* growth differentiation factor-15, *TRANCE* tumor necrosis factor-related activation-induced cytokine, *TRAIL* tumor necrosis factor-related apoptosis-inducing ligand, *CVD* cardiovascular disease, *NPX* normalized protein expression, *adj. p* adjusted p-value for false discovery rate. LogFC (fold change) is reported in log_2_ scale.

A total of 266 different plasma proteins were analyzed with the Cardiovascular II and III, and Inflammation Olink panels. Three proteins were differentially expressed after correction for multiple testing between patients with or without CVD including growth differentiation factor (GDF)-15, tumor necrosis factor (TNF)-related activation-induced cytokine (TRANCE; also named receptor activator of nuclear factor kappa-Β ligand [RANKL]), and TNF-related apoptosis-inducing ligand (TRAIL). Of these, GDF-15 was increased while TRANCE and TRAIL were decreased in patients with CVD (Fig. 1). When results were adjusted for age, sex and BMI, differences in GDF-15 and TRANCE remained significant, while TRAIL was of borderline significance (FDR = 0.055). Additional adjustments for systemic anti-psoriatic treatment did not affect the results. In multivariable linear regression analyses, levels of GDF-15 and TRANCE remained significantly increased and decreased, respectively, after additional adjustment for statin treatment, while TRAIL was no longer significantly decreased in patients with CVD (data not shown).

### Correlation between differentially expressed proteins and subclinical atherosclerosis

**Figure 2:**
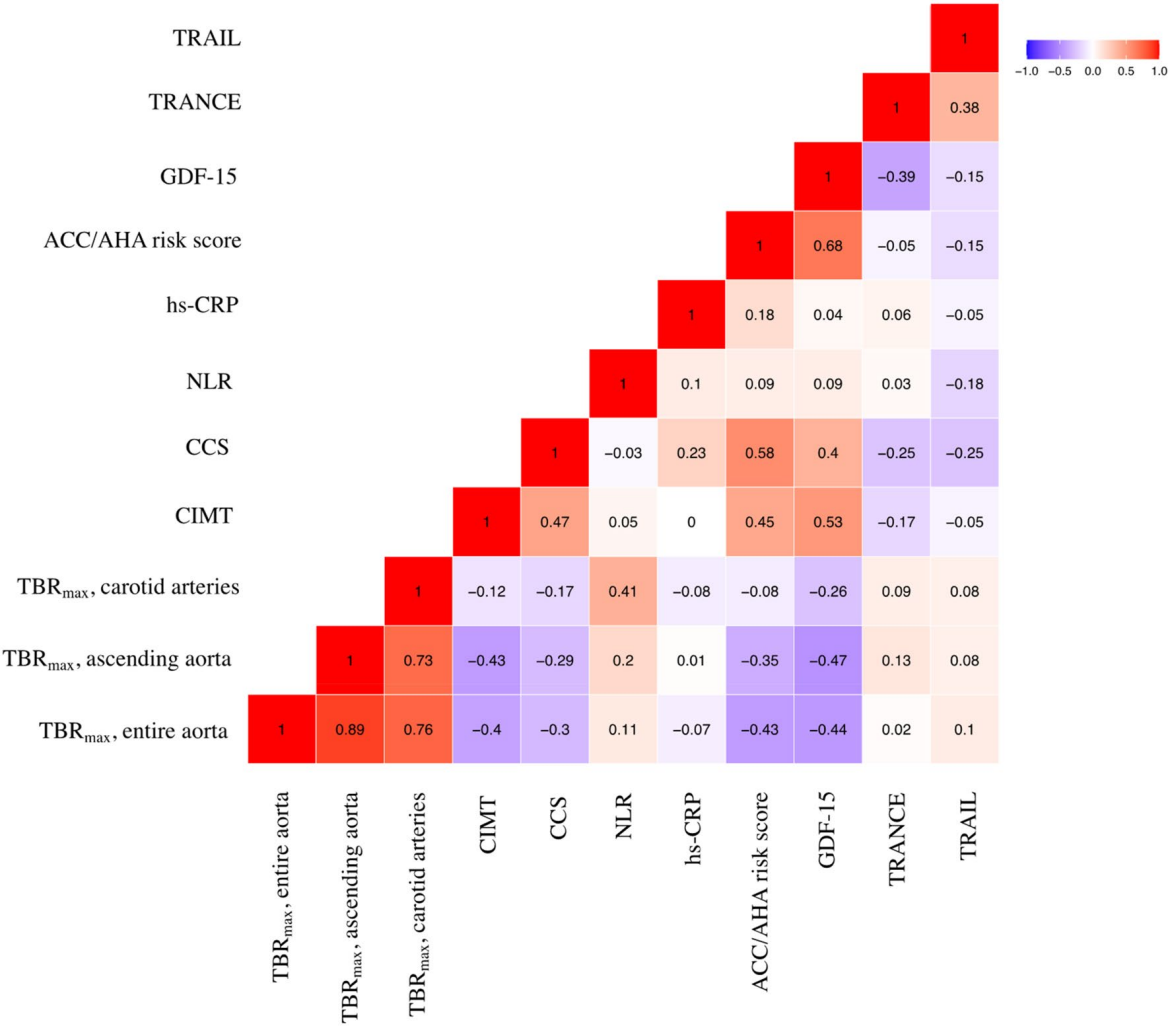
Correlation matrix with Pearson correlation coefficients between differentially expressed proteins, neutrophil-to-lymphocyte ratio (NLR), high-sensitive C-reactive protein (hs-CRP), America College of Cardiology/American Heart Association (ACC/AHA) risk score and markers of subclinical atherosclerosis in patients with psoriasis without cardiovascular disease and statin treatment (n = 36). *TRAIL* tumor necrosis factor-related apoptosis inducing ligand, *TRANCE* tumor necrosis factor-related activation-induced cytokine, *GDF-15* growth differentiation factor-15, *CCS* coronary artery calcium score, *CIMT* carotid intima-media thickness, *TBR*_*max*_ maximum target-to-background ratio.

To investigate whether GDF-15, TRANCE and TRAIL that were found to be linked with clinical atherosclerotic CVD were also associated with subclinical atherosclerosis, we correlated the expression of these three proteins with vascular inflammation determined by FDG uptake in the aorta and carotid arteries, and with CIMT and CCS, respectively, in patients without CVD and statin treatment (n = 36) (Fig. 2). GDF-15 was negatively associated with vascular inflammation in the ascending aorta (r =  − 0.47; p = 0.005) and in the entire aorta (r =  − 0.44; p = 0.010). A positive association was seen between GDF-15 and CIMT (r = 0.53; p < 0.001) and CCS (r = 0.40; p = 0.018). The association between GDF-15 and CIMT was no longer significant after multivariable linear regression analyses with adjustments for ACC/AHA risk score and hs-CRP. GDF-15 was positively associated with the ACC/AHA risk score, which was only available for 32 patients without CVD and statin treatment (r = 0.68, p < 0.001). None of the 3 proteins were associated with hs-CRP or NLR.

**Figure 3:**
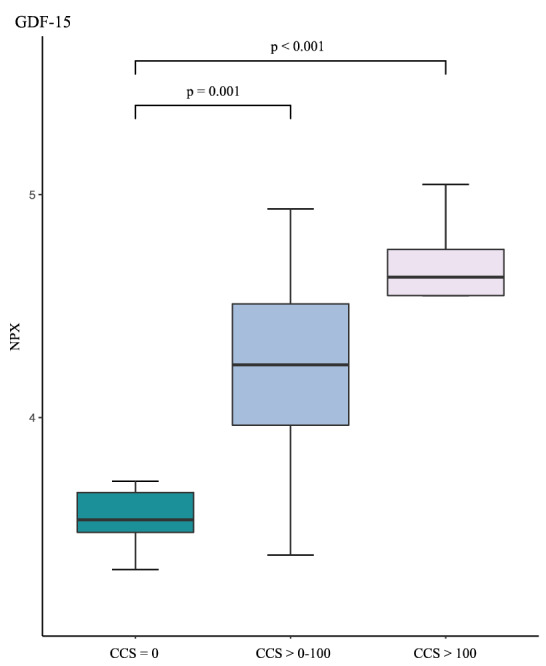
Distribution of growth differentiation factor-15 (GDF-15) between subjects with coronary artery calcium score (CCS) = 0, CCS > 0–100, and CCS > 100 in patients without cardiovascular disease and statin treatment (n = 34). *NPX* normalized protein expression.

Additional analyses showed that GDF-15 was significantly higher in patients with CCS > 100 without CVD and statin treatment (ANOVA; p < 0.001) (Fig. 3). Furthermore, a 10% increase of GDF-15 was associated with CCS > 100 (OR [95% CI] 1.70 [1.34–2.33]; p < 0.001) (Table 2). GDF-15 remained significantly associated with CCS > 100 after adjustments for ACC/AHA risk score and hs-CRP (p = 0.010) (Table 3). TRANCE and TRAIL showed no differences between the three CCS groups (Table 2). GDF-15 was associated with the presence of plaques in both carotid arteries (OR 1.30 [1.09–1.61]; p = 0.007) and was increased in these patients (ANOVA; p = 0.010). TRAIL but not TRANCE was associated with absence of carotid plaques (OR 0.59 [0.39–0.86]; p = 0.010) (Table 2) and was increased in patients with no carotid plaques (ANOVA; p = 0.009) (data not shown).

**Table 2:** Associations between differentially expressed proteins or neutrophil-to-lymphocyte ratio (NLR) and presence of carotid artery plaques and coronary artery calcium score > 100 in patients with psoriasis without cardiovascular disease and statin treatment.

	Carotid artery plaques (n = 36)		Coronary artery calcium score (n = 34)	
	OR (95% CI)	p-value	OR (95% CI)	p-value
GDF-15	1.30 (1.09–1.61)	0.007	1.70 (1.34–2.33)	< 0.001
TRANCE	0.87 (0.70–1.05)	0.154	0.92 (0.75–1.12)	0.388
TRAIL	0.59 (0.39–0.86)	0.010	0.79 (0.55–1.10)	0.169
NLR	1.32 (0.75–2.41)	0.340	1.06 (0.61–1.84)	0.847

For methodological details, see text.

*GDF-15* growth differentiation factor-15, *TRANCE* tumor necrosis factor-related activation-induced cytokine, *TRAIL* tumor necrosis factor-related apoptosis-inducing ligand, *OR* odds ratio, *CI* confidence interval.

**Table 3:** Analyses of growth differentiation factor-15 (GDF-15) and neutrophil-to-lymphocyte ratio (NLR) associations with carotid intima-media thickness, coronary artery calcium score, and vascular inflammation in the carotid arteries, respectively, in patients with psoriasis without cardiovascular disease and statin treatment.

	Carotid intima-media thickness*		
	*β* coefficient	95% CI	p-value
**GDF-15**			
Model 1	0.13	(0.06 to 0.21)	< 0.001
Model 2	0.07	(− 0.05 to 0.18)	0.237
Model 3	0.06	(− 0.05 to 0.18)	0.274

	Coronary artery calcium score**		
	OR	95% CI	p-value
**GDF-15**			
Model 1	1.70	(1.34 to 2.33)	< 0.001
Model 2	1.50	(1.11 to 2.13)	0.013
Model 3	1.56	(1.14 to 2.27)	0.010

	TBR_max_ (carotid arteries)*		
	*β* coefficient	95% CI	p-value
**NLR**			
Model 1	0.13	(0.02 to 0.24)	0.021
Model 2	0.11	(0.01 to 0.22)	0.039
Model 3	0.11	(0.00 to 0.22)	0.044

Model 1, crude results; Model 2, after adjustment for the America College of Cardiology/American Heart Association (ACC/AHA) risk score; Model 3, after adjustment for ACC/AHA risk score and high-sensitivity C-reactive protein.

*TBR*_*max*_ maximum target-to-background ratio, *OR* odds ratio, *CI* confidence interval.

*Linear -and multivariable regression models were performed to assess the association between GDF-15 and NLR and carotid intima-media thickness and vascular inflammation, respectively, in the carotid arteries.

**Ordinal logistic regression was performed to assess the association between GDF-15 and the coronary artery calcium score.

### Correlation between NLR and subclinical atherosclerosis

NLR was positively associated with vascular inflammation in the carotid arteries determined by TBR_max_ (*r* = 0.41; *p* = 0.021). No associations were observed between NLR and vascular inflammation in the ascending aorta and the entire aorta, CIMT and CCS, respectively (Fig. 2). Also, ordinal logistic regression showed no association between NLR and CCS (OR 1.06 [0.61–1.84]; p = 0.847) or carotid artery plaques (OR 1.32 [0.75–2.41]; p = 0.340) in analyses limited to patients without CVD and statin treatment (Table 2). Multivariable linear regression analyses showed that NLR was significantly associated with TBR_max_ (carotid arteries) after adjustments for ACC/AHA risk score (p = 0.039) and when hs-CRP was included in the model (p = 0.044) (Table 3).

## Discussion

This study investigated a large set of plasma proteins related to inflammation and cardiovascular risk, to identify those linked with subclinical atherosclerosis in patients with psoriasis. We first examined differentially expressed proteins in patients with vs. without atherosclerotic CVD and subsequently ascertained if these proteins were linked with diverse measures of subclinical CVD including vascular inflammation, CIMT and carotid plaques, and CCS, in patients without CVD and statin treatment. We found that GDF-15 was increased while TRANCE and TRAIL were decreased in patients with CVD. GDF-15 was negatively associated with vascular inflammation and positively associated with CIMT and CCS in patients without CVD and statin treatment. Additionally, NLR was increased in patients with CVD vs. those without CVD, was positively associated with vascular inflammation in the carotid arteries and showed no association with CIMT and CCS.

Psoriasis is a chronic systemic inflammatory disease linked with increased risk of CVD which is likely mediated, in part, by low grade systemic inflammation^1,3,33^. For example, cumulative exposure to systemic inflammation may promote impaired insulin sensitivity, endothelial dysfunction, increased aortic stiffness, increased CIMT, and vascular inflammation contributing to CVD in patients with psoriasis^3,12,14,34^. While studies of circulating markers of systemic inflammation, e.g. hs-CRP, TNF-α, and interleukin (IL)-6, in psoriasis have been widely reported, fewer reports are available on biomarkers of subclinical CVD in these patients and such studies frequently examined single proteins, e.g. YKL-40 and leptin, and single subclinical CVD endpoints, e.g. endothelial dysfunction or CIMT^17,18,33,35^. Interestingly, however, GlycA, a heterogenous nuclear magnetic resonance signal arising from glycan residues of acute-phase proteins, e.g. α-1-acid glycoprotein (orosomucoid) and α1-antitrypsin, has been linked with subclinical CVD including vascular inflammation and coronary artery disease burden demonstrated by ^18^F-FDG-PET/CT and coronary artery CT angiography, respectively, in patients with psoriasis^15^.

We found that GDF-15 was linked to all examined subclinical CVD measures and that this relationship included both negative (vascular inflammation) and positive (CIMT and CCS) associations. We also found that GDF-15 was positively associated with the ACC/AHA risk score and associated with CCS, but not CIMT, after adjustment for the ACC/AHA risk score and hs-CRP. GDF-15 is a member of the transforming growth factor superfamily that is strongly induced in macrophages by interleukin-1β, TNF-α, and transforming growth factor-β, and is involved in the regulation of multiple biologic functional networks such as inflammatory, apoptotic and angiogenesis pathways^36,37^. For example, increased expression of GDF-15 is observed in atherosclerotic lesions and evidence suggests that plasma GDF-15 is a biomarker of CVD, and an independent negative prognostic marker in diverse patient subsets, e.g. after MI^36–41^. However, experimental studies have indicated that elevated levels of GDF-15 can have protective roles in CVD, e.g. with smaller atherosclerotic lesions observed after transgenic overexpression of GDF-15 in the apoE knockout mouse model of atherosclerosis^42^. A protective role of GDF-15 in atherosclerosis might be compatible with the negative link between GDF-15 levels and subclinical vascular inflammation observed in our study. On the other hand, we found that GDF-15 was increased in patients with psoriasis with prior CVD, which is consistent with results from previous studies of patients without psoriasis^36–41^. We also found a positive association between GDF-15 and the ACC/AHA risk score, and such association has also been observed between GDF-15 and the Framingham risk score in patients with diabetes^43^. Interestingly, additional analyses showed that the levels of GDF-15 remained significant after adjustment for statin treatment, which is in line with previous studies that did not find effects of statins on plasma GDF-15 levels in patients with acute coronary syndrome or diabetes^44–46^. The differing associations observed between GDF-15 and diverse subclinical markers of atherosclerosis in our study add to the on-going discussion of whether GDF-15 is a marker or a maker of CVD^36,37^. Indeed, although the restricted sample size and other limitations of our study do not allow for conclusions, the contradictory links between GDF-15 and subclinical atherosclerotic CVD determined by vascular inflammation compared to CIMT and CCS may suggest that increased GDF-15 in patients with CVD is a consequence rather than a cause of CVD. The divergent results are likely also dependent on varying effectiveness of imaging techniques for detection of inflammation and other characteristics of different atherosclerosis phenotypes in various vascular territories. Notably, evidence has indicated that arterial FDG uptake is not strictly correlated with macrophage accumulation (a conventional hallmark of inflammation) and that comparable FDG uptake is observed in other arterial cells and tissues including medial smooth muscle cells^47^. Our current results should therefore be interpreted with the understanding that the precise pathoanatomic background for results of various noninvasive tests aimed at subclinical atherosclerosis remains to be determined.

We also identified differential expressions of TRANCE and TRAIL in patients with psoriasis with vs. without CVD, and these proteins are involved in psoriasis (arthritis) pathogenesis, bone physiology, and vascular calcification^48–51^. TRANCE and TRAIL are ligands of osteoprotegerin and although these three members of the TNF superfamily have been linked to atherosclerosis, e.g. with plasma levels of osteoprotegerin associated with CVD, and worsened atherosclerosis after genetic deletion of TRAIL in the apoE knockout mouse model, respectively, their effects and underlying mechanisms in atherosclerosis are unclear^48,52^. In our study, osteoprotegerin was not associated with CVD in patients with psoriasis, but TRANCE and TRAIL were decreased in those with CVD, and in patients without CVD and statin treatment, TRAIL was decreased in subjects with carotid artery plaques, and TRAIL and TRANCE showed no relation to CCS. The results for TRAIL are supported by studies indicating that plasma TRAIL levels are decreased in patients with coronary artery disease and associated with worsened prognosis^48^.

NLR is a biomarker of inflammation that is easily obtainable from complete blood counts and which is linked to cardiovascular events and all-cause mortality, and is increased in patients with psoriasis^20,21,53^. In these patients, increased NLR is associated with subclinical CVD, e.g. CIMT and non-calcified coronary artery plaque burden measured by coronary artery CT angiography^22,54^. Furthermore, circulating biomarkers of neutrophil activation may be linked to skin disease severity (e.g. PASI) and vascular inflammation measured by ^18^F-FDG-PET/CT^55^. Indeed, neutrophils play important roles in psoriasis and atherosclerotic CVD^53,56^, but to our knowledge, the association between NLR and vascular inflammation in patients with psoriasis determined by ^18^F-FDG-PET/CT has not been reported previously. Although we only found an association between NLR and inflammation in the carotid arteries (not in the aorta), which remained significant beyond the ACC/AHA risk score, these arteries have previously been recommended as readout vessels that hold the strongest biological validation linking the ^18^F-FDG signal to vascular inflammation^57^. Therefore, this finding supports that NLR could be a useful biomarker of subclinical CVD and contribute to CVD risk stratification in patients with psoriasis. Notably, we did not find associations between NLR and CCS or CIMT, and as noted above, imaging modalities for atherosclerosis differ in their capacity for detection of the predominant (not necessarily strictly coincident) disease processes, e.g. inflammation, neointimal formation, and vascular calcification in different arteries.

The strength of the study was the large-scale proteomic analysis and use of different measures of subclinical atherosclerosis in well-characterised patients with a history of moderate-to-severe psoriasis. However, the sample size was relatively small, a control group without psoriasis was not included and other vascular measures, e.g. endothelial dysfunction and coronary artery disease burden by coronary artery CT angiography were not assessed. Also, our patients had different disease activities and approximately half received systemic anti-psoriatic treatment albeit that adjustment for this treatment did not affect the significance of results for the differentially expressed proteins. Moreover, the study was an observational study that does not allow for causal interferences.

In conclusion, GDF-15 and NLR may serve as biomarkers of subclinical CVD in patients with a history of moderate-to-severe psoriasis. More studies are warranted to define novel biomarkers of CVD risk and their consequences for therapy in these patients.

## Data Availability

The data analyzed in the current study are available from the corresponding author on reasonable request.
